# The impact of severe traumatic brain injury on a novel base deficit- based classification of hypovolemic shock

**DOI:** 10.1186/1757-7241-22-28

**Published:** 2014-04-30

**Authors:** Manuel Mutschler, Ulrike Nienaber, Arasch Wafaisade, Thomas Brockamp, Christian Probst, Thomas Paffrath, Bertil Bouillon, Marc Maegele

**Affiliations:** 1 Department of Trauma and Orthopedic Surgery, Cologne-Merheim Medical Center (CMMC), University of Witten/Herdecke, Ostmerheimer Str. 200, D-51109 Cologne, Germany; 2 Institute for Research in Operative Medicine (IFOM), University of Witten/Herdecke, Ostmerheimer Str. 200, D-51109 Cologne, Germany; 3 Academy for Trauma Surgery, Straße des 17.Juni 106-108, D-10623 Berlin, Germany

**Keywords:** Trauma, Shock, Classification, Vital signs, Base deficit, Transfusion, Traumatic brain injury

## Abstract

**Background:**

Recently, our group has proposed a new classification of hypovolemic shock based on the physiological shock marker base deficit (BD). The classification consists of four groups of worsening BD and correlates with the extent of hypovolemic shock in severely injured patients. The aim of this study was to test the applicability of our recently proposed classification of hypovolemic shock in the context of severe traumatic brain injury (TBI).

**Methods:**

Between 2002 and 2011, patients ≥16 years in age with an AIS_head_ ≥ 3 have been retrieved from the German TraumaRegister DGU® database. Patients were classified into four strata of worsening BD [(class I (BD ≤ 2 mmol/l), class II (BD > 2.0 to 6.0 mmol/l), class III (BD > 6.0 to 10 mmol/l) and class IV (BD > 10 mmol/l)] and assessed for demographic and injury characteristics as well as blood product transfusions and outcomes. The cohort of severely injured patients with TBI was compared to a population of all trauma patients to assess possible differences in the applicability of the BD based classification of hypovolemic shock.

**Results:**

From a total of 23,496 patients, 10,201 multiply injured patients with TBI (AIS_head_ ≥ 3) could be identified. With worsening of BD, a consecutive increase of mortality rate from 15.9% in class I to 61.4% in class IV patients was observed. Simultaneously, injury severity scores increased from 20.8 (±11.9) to 41.6 (±17). Increments in BD paralleled decreasing hemoglobin, platelet counts and Quick’s values. The number of blood units transfused correlated with worsening of BD. Massive transfusion rates increased from 5% in class I to 47% in class IV. Between multiply injured patients with TBI and all trauma patients, no clinically relevant differences in transfusion requirement or massive transfusion rates were observed.

**Conclusion:**

The presence of TBI has no relevant impact on the applicability of the recently proposed BD-based classification of hypovolemic shock. This study underlines the role of BD as a relevant clinical indicator of hypovolaemic shock during the initial assessment in respect to haemostatic resuscitation and transfusion requirements.

## Background

Uncontrolled haemorrhage is still responsible for the half of all trauma-related deaths within the first 48 hours after hospital admission [1-3]. This underlines the pivotal role of the early recognition and treatment of hypovolaemia in acute trauma care. The Advanced Trauma Life Support (ATLS) proclaims for the initial evaluation of circulatory depletion four classes of hypovolemic shock based upon an estimated blood loss in percent and corresponding vital signs [4]. However, recent analyses on data of multiple injured patients derived from the TraumaRegister DGU® and the TARN (Trauma Audit and Research Network) database indicated that the current ATLS classification of hypovolemic shock displays substantial deficits in the initial assessment of multiply injured trauma patients [5-8].

Based on these observations, we developed and validated a new classification of hypovolemic shock on the physiological shock marker base deficit (Table 1) [9]. The defined four strata of worsening BD indicated the acute presence of hypovolemic shock related to the need for haemostatic resuscitation, transfusion requirement, laboratory findings and outcomes [9]. This new classification of hypovolemic shock predicted transfusion requirements and mortality even more appropriately than the current ATLS classification of hypovolemic shock [9].

**Table 1:** **The BD-based classification of hypovolemic shock **[9]

	**Class I**	**Class II**	**Class III**	**Class IV**
Shock	none to minimal	mild	moderate	severe
BD at admission (mmol/l)	≤ 2	> 2.0 to 6.0	> 6.0 to 10.0	> 10.0
Need for blood products	watch	consider	act	Be prepared for massive transfusion

*Abbreviations: BD* base deficit.

More than 40% of all trauma patients captured in the TraumaRegister DGU® sustain a combination of injuries including severe traumatic brain injury (TBI) [8]. Together with hemorrhage, TBI is the leading cause of death in multiply injured trauma patients [1-3,10] and the coincidence of TBI and hemorrhage has been associated with an even worse overall morbidity and mortality [11]. Several studies have shown that TBI may impair cardiovascular compensation to acute blood loss [12-16]. In detail, it has been demonstrated that the autonomic response to haemorrhage is disturbed in the presence of TBI and the physiological response to acute blood loss, like hypotension or the modification of vascular tone may be impaired or delayed [14,15,17]. Yuan and colleagues even demonstrated that TBI may suppress spontaneous hemodynamic recovery from hemorrhage and also impede the efficacy of fluid resuscitation [12,18]. Additionally, traditional vital signs like heart rate (HR), systolic blood pressure (SBP), shock index (SI) and Glasgow Coma Scale may be affected, indicating that current assessment strategies, like the ATLS classification of hypovolemic shock, have to be used with caution [8,14,19].

Therefore, the aim of this study was to test the applicability of the novel BD-based classification of hypovolemic shock in the context of severe traumatic brain injury (TBI).

## Methods

### The TraumaRegister DGU® of the German Trauma Society

The TraumaRegister DGU®, founded in 1993, is the official trauma registry of the German Trauma Society. Details of this registry have been published *in extenso* elsewhere [5,8,9,20]. To date, datasets from approximately > 450 hospitals have been entered into the database. The TraumaRegister DGU® captures all severe trauma patients, who are either admitted to the hospital via the emergency department (ED) with subsequent admission to an intensive care unit (ICU)/intermediate care unit (IMC) or reach the hospital with vital signs and die prior to ICU/IMC admission. The TraumaRegister DGU® is approved by the review board of the German Trauma Society (DGU) and is in compliance with the institutional requirements of its members. Furthermore, all data analyses have been approved by the review board of the “Sektion NIS” of the German Trauma Society.

### Data analyses

Datasets of multiple injured patients entered into the TraumaRegister DGU® between 2002 and 2011 were analyzed. Inclusion criteria were age ≥ 16 years, primary admission, and complete datasets for base deficit upon admission blood gas analysis in the emergency department. Severe traumatic brain injury was defined by an AIS_head_ ≥ 3 (abbreviated injury scale) as previously described [21-23]. Based on these criteria, 10,201 patients with severe TBI could be identified and were classified into four strata of worsening BD according to the recently proposed classification of hypovolemic shock (Table 1) [9]. Assessments included demographics, injury patterns and vital signs as present upon ED arrival. Additionally, therapeutic interventions such as administration of blood products, intravenous fluids and vasopressors were analyzed. The assessment of mortality was performed by analyzing the overall in-hospital mortality. Massive transfusion (MT) was defined by the administration of ≥ 10 blood products between ED arrival and ICU admission. Coagulopathy was defined by a Quick’s value ≤ 70%, which is equivalent to an international normalized ratio (INR) of approximately 1.3 [22,24]. For the evaluation of the applicability of the novel BD-based classification in the context of severe TBI, patients with an AIS_head_ ≥ 3 were classified according to their BD at ED admission and compared to both, an unselected cohort of all trauma patients derived from the TraumaRegister DGU® as well as to patients without a significant TBI (no TBI). Subsequently, transfusion requirements within the different cohorts were assessed.

### Statistical methods

Data are shown as mean ± standard deviation (SD) for continuous variables or percentages (%) for categorical variables. GCS is displayed as median and interquartile ranges (IQR). For continuous variables, normal distribution was excluded using the Shapiro-Wilk test. To detect differences between the four groups of worsening BD a Kruskal-Wallis test was performed. Categorical variables were analyzed accordingly with the Chi-square test. For all statistical analyses, a probability of less than 0.05 was considered to be statistically significant. All data were analyzed using IBM SPSS (IBM SPSS 19, Chicago, USA).

## Results

### Characteristics of the four classes of hypovolemic shock in TBI patients

A total of 23,496 patients from the TraumaRegister DGU® were identified for further analysis. Out of these, 10,201 (43.4%) patients presented with severe TBI defined by an AIS_head_ ≥ 3. General demographics and trauma mechanism for the four classes of hypovolemic shock are shown in Table 2. Worsening of BD category was associated with an increased injury severity score (ISS 25.8 ± 11.9 in class I to 41.6 ± 17.0 in class IV). Through the classes I to IV, this was paralleled by an increased incidence of sepsis (7.5% to 14.1%) and multiple organ failure (18% to 44.4). Overall in-hospital mortality increased from 15.9% in class I patients to 61.4% in class IV patients (Table 3). No relevant tachycardia was observed in any group and a substantial hypotension was found in class IV patients only. Patients with a BD > 10.0 mmol/l (class IV) were coagulopathic (Table 4) and presented with significantly decreased haemoglobin levels (9.4 ± 3.2 g/dl) and platelet counts (177 ± 93 tsd/μl) compared to class I patients (haemoglobin 12.6 ± 2.3 g/dl; platelet counts 208 ± 72 tsd/μl).

**Table 2:** Patients classified by BD (classes I to IV): demographics, injury mechanism and severities as well as outcome parameters

	**Class I**	**Class II**	**Class III**	**Class IV**
	**BD ≤ 2.0 (none to minimal)**	**BD >2.0 to 6.0 (mild)**	**BD > 6.0 to 10.0 (moderate)**	**BD > 10.0 (severe)**
**Demographics**				
n (total, %)	4369 (42.8)	3658 (35.9)	1404 (13.8)	770 (7.5)
Male (n, %)	3211 (73.9)	2636 (72.6)	982 (70.5)	523 (68.2)
Age (years; mean ± SD)	50.9 (21.4)	46.4 (21)	45.6 (20.6)	46.2 (20.3)
Blunt trauma (n, %)	4121 (97.3)	3467 (96.8)	1332 (96.2)	703 (93.1)
**Injury severity**				
ISS (points; mean ± SD)	25.8 (11.9)	30.2 (13.6)	35.2 (15.9)	41.6 (17.0)
AIS Thorax ≥3 points (n; %)	1476 (33.8)	1642 (44.9)	742 (52.8)	486 (63.1)
AIS Abdomen ≥3 points (n; %)	236 (5.4)	395 (10.8)	270 (19.2)	19.9 (25.8)
AIS Pelvis/Extremities ≥3 points (n; %)	650 (14.9)	892 (24.4)	469 (33.4)	305 (39.6)
**Outcome**				
Mortality (n; %)	696 (15.9)	805 (22)	502 (35.8)	473 (61.4)
Hospital LOS (days; mean ± SD)	18.1 (17.8)	21.7 (25)	20.8 (24.8)	16 (29.1)
ICU (days; mean ± SD)	10.8 (12.2)	13.5 (13.8)	14 (15.9)	11.1 (16.8)
Ventilator days (days; mean ± SD)	7.3 (10.4)	9.7 (11.8)	10.5 (13.1)	9.1 (14.4)
MOF (n; %)	694 (18)	876 (26.8)	389 (32.6)	256 (44.4)
Sepsis (n, %)	296 (7.5)	428 (12.5)	194 (15.8)	83 (14.1)

(n = 10,201; p < 0.001 for all parameters).

*Abbreviations: ISS* injury severity score, *AIS* abbreviated injury scale, *LOS* length of stay, *ICU* intensive care unit, *MOF* multiple organ failure.

**Table 3:** Patients classified by BD (classes I to IV): traditional vital signs as presented at ED admission and at scene

	**Class I**	**Class II**	**Class III**	**Class IV**
	**BD ≤ 2.0 (none to minimal)**	**BD >2.0 to 6.0 (mild)**	**BD > 6.0 to 10.0 (moderate)**	**BD > 10.0 (severe)**
**Vital signs**				
SBP at ED (mmHg; mean ± SD)	132.3 (28.6)	124.3 (30)	113.2 (32.4)	95.7 (40.9)
HR at ED (beats/min; mean ± SD)	84.3 (18.4)	88.7 (21.6)	95.3 (23.4)	96.5 (32.9)
GCS at ED (points; median, IQR)	3 (3–14)	3 (3–8)	3 (3–3)	3 (3–3)
Intubation rate [pre-hospital] (n; %)	2306 (54.5)	2563 (71.9)	1116 (81.4)	674 (89.2)

(n = 10,201; p < 0.001 for all parameters).

*Abbreviations: SBP* systolic blood pressure, *HR* heart rate, *GCS* Glasgow coma scale, *ED* Emergency Department.

**Table 4:** Patients classified by BD (classes I to IV): laboratory findings

	**Class I**	**Class II**	**Class III**	**Class IV**
	**BD ≤ 2.0 (none to minimal)**	**BD >2.0 to 6.0 (mild)**	**BD > 6.0 to 10.0 (moderate)**	**BD > 10.0 (severe)**
**Laboratory findings**				
Haemoglobin (g/dl; mean ± SD)	12.6 (2.3)	11.8 (2.5)	10.7 (2.9)	9.4 (3.2)
Thrombocytes (tsd/μl; mean ± SD)	208 (72)	204 (70)	188 (73)	177 (93)
Quick’s value (%; mean ± SD)	84 (20.9)	77.8 (22.8)	68.2 (25.0)	54.7 (26.6)
aPTT (seconds; mean ± SD)	30.7 (11)	33.9 (16.3)	41.9 (26.6)	61.8 (42.8)

(n = 10,201; p < 0.001 for all parameters).

*Abbreviations: aPTT* activated partial thromboplastin time*.*

Fluid resuscitation and transfusion requirement in correlation to increasing BD category are demonstrated in Table 5. Patients of class I received 1.5 ± 6 blood units, whereas class IV patients received 15.1 ± 22 blood units until ICU admission. The average transfusion ratio between packed red blood cells (pRBC’s) and fresh frozen plasma (FFP’s) was almost 1:1 within all groups. Likewise, observed and predicted transfusion requirements were concordant as the number of blood products transfused paralleled increased TASH (Trauma-Associated Severe Hemorrhage) scores.

**Table 5:** Patients classified by BD (classes I to IV): haemostatic and fluid resuscitation

	**Class I**	**Class II**	**Class III**	**Class IV**
	**BD ≤ 2.0 (none to minimal)**	**BD >2.0 to 6.0 (mild)**	**BD > 6.0 to 10.0 (moderate)**	**BD > 10.0 (severe)**
**Transfusion requirements**				
All blood products/units (n; mean ± SD)	1.5 (6)	4.2 (11.5)	9.4 (17.5)	15.1 (22)
pRBC transfusions/units (n; mean ± SD)	1.1 (3.4)	2.6 (5.8)	5 (8.4)	8.1 (10.9)
FFP transfusions/units (n; mean ± SD)	0.8 (3.1)	2.3 (11.6)	4.1 (7.4)	6.2 (10)
TC transfusion/units (n; mean ± SD)	0.1 (0.5)	0.3 (1.1)	0.6 (1.8)	0.9 (1.8)
TASH Score (points; mean ± SD)	3.1 (3.1)	5.6 (4.0)	10.0 (5.0)	13.5 (5.3)
IV fliuds at ED (ml; mean ± SD)	1498 (1531)	2143 (2075)	2573 (2404)	2964 (2564)
Vasopressors at ED (n; %)	888 (21.9)	1233 (36.1)	690 (52.3)	549 (75.8)

(n = 10,201; p < 0.001 for all parameters).

*Abbreviations: pRBC* packed red blood cells, *FFP* fresh frozen plasma, *TC* thrombocyte concentrate, *TASH* Trauma-associated severe hemorrhage score, *i.v.* intravenous, *ED* Emergency Department.

### Comparison of transfusion requirement between multiply injured patients with TBI and all trauma patients classified according to the BD-based classification of hypovolemic shock

**Figure 1:**
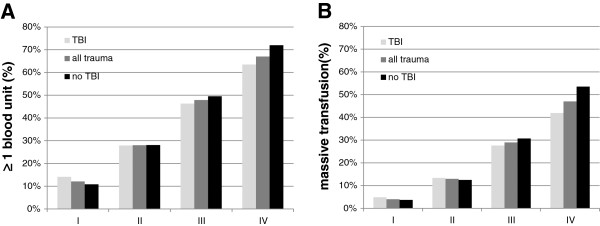
**Transfusion requirements in multiply injured patients with TBI, all trauma patients and multiply injured trauma patients without TBI. A)** Percent of patients with ≥ 1 blood product. (n = 23,496); **B)** Percent of patients with massive transfusion (≥10 blood units until ICU admission). (n = 23,496). *Abbreviations: TBI: traumatic brain injury.*

In multiply injured patients with TBI, an increase in BD category was associated with a progressively increasing transfusion requirement (Figure 1). The percentage of patients who received ≥ 1 blood unit during early ED resuscitation increased from 14% in class I to 64% in class IV (Figure 1A), while simultaneously massive transfusion rates increased stepwise from 5% in class I to 42% in class IV (Figure 1B). However, substantial and clinical relevant differences between patients who sustained TBI and the general trauma population were not observed within the four classes of hypovolemic shock based on BD. Similar results were seen when multiply injured patients with TBI were compared to a cohort of severely injured patients without TBI (Figure 1A and B). Only in group IV, patients without TBI had a slightly higher overall transfusion requirement. However, the clinical relevance of this finding remains questionable.

## Discussion

The aim of this study was to test the applicability of the recently proposed classification of hypovolemic shock based on BD in multiple injured patients who have sustained severe traumatic brain injury.

Previous studies have demonstrated that base deficit detects the presence of hypovolemic shock and correlates with the need for haemostatic resuscitation, transfusion requirements, laboratory findings as well as mortality rates. In times of point-of-care testing, BD is available within minutes after ED admission [25]. Recent research provides evidence that the assessment of BD is a more useful approach to quantify the extent of hypovolemic shock than the estimation of blood loss, the extent of volume resuscitation or traditional vital signs such as HR and SBP [9,26-29] and may help to distinguish major from minor trauma [30]. The four classes of hypovolemic shock based on BD has been compared and validated against the ATLS classification of hypovolaemic shock. ATLS serves as a standard protocol for the initial assessment and treatment of severely injured trauma patients and has been widely implemented over the past decades. In this analysis, the BD-based classification has been shown to predict transfusion requirements and mortality more appropriately than the current ATLS classification of hypovolemic shock [9].

The present study focused on TBI patients with an AIS_head_ ≥ 3. Within this group of patients, worsening of BD category was associated with an increased injury severity and mortality, which is in accordance with several previous analyses [9,31-34] and has been also observed in pediatric and elderly trauma populations [35-38]. As expected, mortality rates across the four classes of hypovolemic shock were higher in multiple injured patients with TBI. In particular, 15.9% of class I and 22% of class II patients died within hospital stay and therefore nearly doubled mortality compared to our previous analysis on a general trauma population [9]. These findings are substantiated by Tremblay and colleagues who demonstrated that mortality increases with successive increase in base deficit but is also highly dependent on injury pattern. In TBI patients, mortality was markedly higher at a given BD than in patients suffering from stab wounds or lacerations [39]. Furthermore, in young trauma patients without TBI, a 25% mortality rate was observed at a BD level of 15 mmol/l. In contrast, TBI patients showed same mortality rates already at a BD level of 8 mmol/l [33].

In the present study, increasing BD was associated with signs of hemodynamical instability and laboratory alterations such as drops in hemoglobin levels and platelet counts. Approximately one out of four patients in class III and IV presented coagulopathic at ED arrival which is in accordance to a previous analysis on isolated traumatic brain injuries by Wafaisade and colleagues [22]. Furthermore, BD correlated with an increasing overall amount of transfused blood units and with an increasing risk of ongoing hemorrhage as reflected by increasing TASH (trauma-associated severe hemorrhage) score values. These observations are supported by several studies demonstrating an association between increasing BD and the increased need for blood product transfusions as well as increasing amounts of i.v. fluids [9,31,32,39].

In order to further assess the applicability of the BD-based classification of hypovolemic shock in the context of severe TBI, multiple injured patients who sustained also brain injury were compared to a cohort of all trauma patients. As there has been no gold standard introduced yet to assess hypovolaemic shock including blood loss in trauma which triggers therapeutic measures and interventions, the authors have decided to analyze and compare for transfusion requirements as a well-established surrogate for hypovolaemia. Both, the percentage of patients who received ≥ 1 blood product as well as the percentage of patients who received massive transfusions were increasing throughout the groups I to IV. However, a relevant difference between both patient cohorts was not observed at all. In a second step, severely injured patients with TBI were also compared to a cohort of multiply injured trauma patients without any significant TBI. Only in group IV, a slight difference in transfusion requirements between both groups was observed. However, the clinical relevance of this finding remains questionable. These results are substantiated by an analysis on 131 patients with isolated head injury, demonstrating that TBI does neither alter BD levels nor reliably predicts the presence of TBI [23]. Therefore, the authors concluded that in patients with isolated head injury an increase of BD should lead to a careful assessment of extracranial injuries as the underlying reason for hypoperfusion and hence the raise in BD.

Some limitations of the present study have to be acknowledged. First, this analysis is a retrospective study of trauma registry data with all the shortcomings associated with such an analysis. Second, not every ED is equipped with point-of-care testing. Consequently, BD values may not be available within the first minutes after ED admission. Further prospective clinical trials should be conducted to assess the accuracy of the proposed classification of hypovolemic shock in the clinical setting. But in spite of these restrictions, we are confident that determination of base deficit, as proclaimed in the BD based classification of hypovolemic shock, is a useful clinical tool to early risk-stratify patients who are in a state of hypovolemic shock and in need for early blood product transfusions, also in patients who sustained traumatic brain injury.

## Conclusions

Our recently proposed classification of hypovolemic shock based on admission base deficit has been tested for its applicability in a large cohort of multiple injured patients with accompanying TBI. Within the four classes of hypovolemic shock (class I to IV), no clinical relevant differences in transfusion requirement in multiple injured patients with TBI and all trauma patients was observed. Consequently, this study underlines the role of BD as a relevant clinical indicator of hypovolaemic shock during the initial assessment and as a guide for transfusion requirements also in patients with traumatic brain injury.

## Abbreviations

AIS: Abbreviated injury scale; ATLS: Advanced Trauma Life Support; BD: Base deficit; DGU: Deutsche Gesellschaft für Unfallchirurgie (German Trauma Society); ED: Emergency Department; FFP: Fresh frozen plasma; GCS: Glasgow coma scale; HR: Heart rate; ICU: Intensive care unit; INR: International normalized ratio; IQR: Interquartile ranges; ISS: Injury severity score; i.v.: Intravenous; LOS: Length of stay; MOF: Multiple organ failure; MT: Massive transfusion; PC: Platelet concentrate; POCT: Point of care testing; pRBC: Packed red blood cells; PT: Prothrombin time; SBP: Systolic blood pressure; TARN: Trauma Audit and Research Network; TASH: Trauma-associated severe hemorrhage score; TBI: Traumatic brain injury.

## Competing interests

The authors declare that they have no competing interests. This is an unfunded study.

## Authors’ contributions

MMutschler contributed to study design, acquisition of data, interpretation and recording of paper. UN and BB contributed to analysis and interpretation of data and revision of the article. TB, AW, CP and TP contributed to study design and revision of the article. MMaegele contributed to study conception and design, acquisition of data, analysis and interpretation of data, and revision of the article. All authors read and approved the final manuscript.

## The TraumaRegister DGU

Committee of Emergency Medicine, Intensive Care and Trauma Management of the DGU (Sektion NIS).

## References

[B1] Anderson ID Woodford M De Dombal FT Irving M Retrospective study of 1000 deaths from injury in England and Wales Br Med J 1988 296 1305 1308 10.1136/bmj.296.6632.1305 3133060 PMC2545772

[B2] Evans JA Van Wessem KJP McDougall D Lee KA Lyons T Balogh ZJ Epidemiology of traumatic deaths: comprehensive population-based assessment World J Surg 2010 34 158 163 10.1007/s00268-009-0266-1 19882185

[B3] Sauaia A Moore FA Moore EE Moser KS Brennan R Read RA Pons PT Epidemiology of trauma deaths: a reassessment J Trauma 1995 38 185 193 10.1097/00005373-199502000-00006 7869433

[B4] Kortbeek JB Al Turki S Ali J Antoine J Bouillon B Brasel K Brenneman F Brink PR Brohi K Burris D Burton R Chapleau W Cioffi W Collet E Cooper A Cortes J Eskesen V Fildes J Gautam S Gruen RL Gross R Hansen KS Henny W Hollands MJ Hunt RC Jover Navalon JM Kaufmann CR Knudson P Koestner A Kosir R Advanced trauma life support, 8th edition, the evidence for change J Trauma 2008 64 1638 1650 10.1097/TA.0b013e3181744b03 18545134

[B5] Mutschler M Nienaber U Brockamp T Wafaisade A Wyen H Peiniger S Paffrath T Bouillon B Maegele M A critical reappraisal of the ATLS classification of hypovolaemic shock: does it really reflect clinical reality? Resuscitation 2012 84 309 313 22835498 10.1016/j.resuscitation.2012.07.012

[B6] Guly HR Bouamra O Little R Dark P Coats T Driscoll P Lecky FE Testing the validity of the ATLS classification of hypovolaemic shock Resuscitation 2010 81 1142 1147 10.1016/j.resuscitation.2010.04.007 20619954

[B7] Guly HR Bouamra O Spiers M Dark P Coats T Lecky FE Vital signs and estimated blood loss in patients with major trauma: testing the validity of the ATLS classification of hypovolaemic shock Resuscitation 2011 82 556 559 10.1016/j.resuscitation.2011.01.013 21349628

[B8] Mutschler M Nienaber U Munzberg M Fabian T Paffrath T Wolfl C Bouillon B Maegele M Assessment of hypovolaemic shock at scene: is the PHTLS classification of hypovolaemic shock really valid? Emerg Med J 2014 31 35 40 10.1136/emermed-2012-202130 23302502

[B9] Mutschler M Nienaber U Brockamp T Wafaisade A Fabian T Paffrath T Bouillon B Maegele M Dgu Renaissance of base deficit for the initial assessment of trauma patients: a base deficit-based classification for hypovolemic shock developed on data from 16,305 patients derived from the TraumaRegister DGU Crit Care 2013 17 R42 10.1186/cc12555 23497602 PMC3672480

[B10] Bruns J Hauser WA The epidemiology of traumatic brain injury: a review Epilepsia 2003 44 Suppl 1 2 10 14511388 10.1046/j.1528-1157.44.s10.3.x

[B11] Jeremitsky E Omert L Dunham CM Protetch J Rodriguez A Harbingers of poor outcome the day after severe brain injury: hypothermia, hypoxia, and hypoperfusion J Trauma 2003 54 312 319 10.1097/01.TA.0000037876.37236.D6 12579057

[B12] Yuan XQ Wade CE Clifford CB Suppression by traumatic brain injury of spontaneous hemodynamic recovery from hemorrhagic shock in rats J Neurosurg 1991 75 408 414 10.3171/jns.1991.75.3.0408 1651379

[B13] Yuan XQ Wade CE Influences of traumatic brain injury on the outcomes of delayed and repeated hemorrhages Circ Shock 1991 35 231 236 1777959

[B14] Goldstein B Toweill D Lai S Sonnenthal K Kimberly B Uncoupling of the autonomic and cardiovascular systems in acute brain injury Am J Physiol 1998 275 R1287 R1292 9756562 10.1152/ajpregu.1998.275.4.R1287

[B15] McMahon CG Kenny R Bennett K Kirkman E Modification of acute cardiovascular homeostatic responses to hemorrhage following mild to moderate traumatic brain injury Crit Care Med 2008 36 216 224 10.1097/01.CCM.0000295425.41831.85 18090349

[B16] McMahon CG Kenny R Bennett K Little R Kirkman E Effect of acute traumatic brain injury on baroreflex function Shock 2011 35 53 58 10.1097/SHK.0b013e3181e687c6 20458265

[B17] Chen B Mutschler M Yuan Y Neugebauer E Huang Q Maegele M Superimposed traumatic brain injury modulates vasomotor responses in third-order vessels after hemorrhagic shock Scand J Trauma Resusc Emerg Med 2013 21 77 10.1186/1757-7241-21-77 24257108 PMC3843561

[B18] Yuan XQ Wade CE Traumatic brain injury attenuates the effectiveness of lactated Ringer’s solution resuscitation of hemorrhagic shock in rats Surg Gynecol Obstet 1992 174 305 312 1553610

[B19] McMahon CG Kenny R Bennett K Little R Kirkman E The effect of acute traumatic brain injury on the performance of shock index J Trauma 2010 69 1169 1175 10.1097/TA.0b013e3181cc8889 20571456

[B20] Scoring study committee of the German Society of Trauma Surgery Trauma register of the German Society of Trauma Surgery Unfallchirurgie 1994 97 230 237 8197471

[B21] Grote S Böcker W Mutschler W Bouillon B Lefering R Diagnostic value of the Glasgow Coma Scale for traumatic brain injury in 18,002 patients with severe multiple injuries J Neurotrauma 2011 28 527 534 10.1089/neu.2010.1433 21265592

[B22] Wafaisade A Lefering R Tjardes T Wutzler S Simanski C Paffrath T Fischer P Bouillon B Maegele M Acute Coagulopathy in Isolated Blunt Traumatic Brain Injury Neurocrit Care 2010 12 211 219 10.1007/s12028-009-9281-1 19806475

[B23] Zehtabchi S Sinert R Soghoian S Liu Y Carmody K Shah L Kumar M Lucchesi M Identifying traumatic brain injury in patients with isolated head trauma: are arterial lactate and base deficit as helpful as in polytrauma? Emerg Med J 2007 24 333 335 10.1136/emj.2006.044578 17452699 PMC2658477

[B24] Wafaisade A Wutzler S Lefering R Tjardes T Banerjee M Paffrath T Bouillon B Maegele M Drivers of acute coagulopathy after severe trauma: a multivariate analysis of 1987 patients Emerg Med J 2010 27 934 939 10.1136/emj.2009.088484 20515913

[B25] Mutschler M Brockamp T Wafaisade A Lipensky A Probst C Bouillon B Maegele M “Time to TASH”: how long does complete score calculation take to assess major trauma hemorrhage? Transfus Med 2013 doi:10.1111/tme.12089 10.1111/tme.12089 24283469

[B26] Rixen D Siegel JH Bench-to-bedside review: oxygen debt and its metabolic correlates as quantifiers of the severity of hemorrhagic and post-traumatic shock Crit Care 2005 9 441 453 10.1186/cc3526 16277731 PMC1297598

[B27] Brasel KJ Guse C Gentilello LM Nirula R Heart rate: is it truly a vital sign? J Trauma 2007 62 812 817 10.1097/TA.0b013e31803245a1 17426534

[B28] Jansen TC Van Bommel J Mulder PG Rommes JH Schieveld SJM Bakker J The prognostic value of blood lactate levels relative to that of vital signs in the pre-hospital setting: a pilot study Crit Care 2008 12 R160 10.1186/cc7159 19091118 PMC2646325

[B29] Parks JK Elliott AC Gentilello LM Shafi S Systemic hypotension is a late marker of shock after trauma: a validation study of Advanced Trauma Life Support principles in a large national sample Am J Surg 2006 192 727 731 10.1016/j.amjsurg.2006.08.034 17161083

[B30] Paladino L Sinert R Wallace D Anderson T Yadav K Zehtabchi S The utility of base deficit and arterial lactate in differentiating major from minor injury in trauma patients with normal vital signs Resuscitation 2008 77 363 368 10.1016/j.resuscitation.2008.01.022 18367305

[B31] Davis JW Parks SN Kaups KL Gladen HE O’Donnell-Nicol S Admission base deficit predicts transfusion requirements and risk of complications J Trauma 1996 41 769 774 10.1097/00005373-199611000-00001 8913202

[B32] Rixen D Raum M Bouillon B Lefering R Neugebauer E Base deficit development and its prognostic significance in posttrauma critical illness: an analysis by the trauma registry of the Deutsche Gesellschaft für unfallchirurgie Shock 2001 15 83 89 11220646 10.1097/00024382-200115020-00001

[B33] Rutherford EJ Morris JA Reed GW Hall KS Base deficit stratifies mortality and determines therapy J Trauma 1992 33 417 423 10.1097/00005373-199209000-00014 1404512

[B34] Siegel JH Rivkind AI Dalal S Goodarzi S Early physiologic predictors of injury severity and death in blunt multiple trauma Arch Surg 1990 125 498 508 10.1001/archsurg.1990.01410160084019 2322117

[B35] Jung J Eo E Ahn K Cheon Y Initial base deficit as predictors for mortality and transfusion requirement in the severe pediatric trauma except brain injury Pediatr Emerg Care 2009 25 579 581 10.1097/PEC.0b013e3181b9b38a 19755892

[B36] Kincaid EH Chang MC Letton RW Chen JG Meredith JW Admission base deficit in pediatric trauma: a study using the National Trauma Data Bank J Trauma 2001 51 332 335 10.1097/00005373-200108000-00018 11493795

[B37] Randolph LC Takacs M Davis KA Resuscitation in the pediatric trauma population: admission base deficit remains an important prognostic indicator J Trauma 2002 53 838 842 10.1097/00005373-200211000-00006 12435932

[B38] Davis JW Kaups KL Base deficit in the elderly: a marker of severe injury and death J Trauma 1998 45 873 877 10.1097/00005373-199811000-00005 9820695

[B39] Tremblay LN Feliciano DV Rozycki GS Assessment of initial base deficit as a predictor of outcome: mechanism of injury does make a difference Am Surg 2002 68 689 693 12206603

